# Präventive Hausbesuche als Intervention zur Gesundheitsförderung älterer Menschen

**DOI:** 10.1007/s00103-026-04286-8

**Published:** 2026-08-13

**Authors:** Britta Blotenberg, Anne Gebert

**Affiliations:** 1 FB Wirtschaft, Institut für eHealth, Management und Versorgung im Gesundheitswesen (IeMVG), Hochschule Flensburg, Kanzleistraße 91–93, 24943 Flensburg, Deutschland; 2 Deutsches Institut für angewandte Pflegeforschung (DIP), Köln, Nordrhein-Westfalen Deutschland

**Keywords:** Präventive Hausbesuche, Pflegebedürftigkeit, Gesundheit, Selbstmanagement, Demografischer Wandel, Community care, Elderly, Health promotion, Health service, Preventive home visit, Demographic change

## Abstract

In diesem Diskussionsbeitrag werden Präventive Hausbesuche (PHB) als niedrigschwellige, aufsuchende Beratungs- und Unterstützungsangebote zur Gesundheitsförderung älterer Menschen diskutiert. PHB können dazu beitragen, Risiken wie Sturzgefahr, soziale Isolation oder Ernährungsmängel frühzeitig zu erkennen, die Gesundheits- und Versorgungskompetenz zu stärken und selbstständige Lebensführung im häuslichen Umfeld zu unterstützen. In Deutschland existiert eine wachsende Zahl von Projekten, deren Konzepte sich hinsichtlich Zielgruppen, Zugangswege, Qualifikation der Besuchskräfte, Leistungsspektrum, Evaluation, Trägerschaft und Finanzierung deutlich unterscheiden. Die Analyse zeigt, dass PHB an der Schnittstelle von kommunaler Altenhilfe, Gesundheitswesen und Pflege angesiedelt sind. Eine zentrale Herausforderung besteht in der langfristigen Finanzierung und Verstetigung bislang vielfach projektförmig organisierter Angebote. Für eine nachhaltige Implementierung sind konzeptionelle Klärungen, belastbare Evaluationen sowie sektorübergreifende Struktur- und Finanzierungsmodelle erforderlich. Die Prävention von Pflegebedürftigkeit sollte gesundheitspolitisch gezielter fokussiert und strukturell stärker in der gesetzlichen Kranken- und Pflegeversicherung verankert werden.

## Abstract (en)

This discussion paper examines preventive home visits (PHVs) as low-threshold, outreach-based counselling and support services aimed at promoting the health of older people. PHVs can help to identify risks such as the risk of falls, social isolation or nutritional deficiencies at an early stage, strengthen health and care literacy, and support independent living in the home environment. In Germany, there is a growing number of projects whose approaches differ significantly in terms of target groups, access routes, the qualifications of visiting staff, the range of services, evaluation, organisational structure and funding. The analysis shows that PHVs are situated at the interface between local authority support for older people, the health service and long-term care. A key challenge lies in the long-term funding and sustainability of services that have so far often been organised on a project basis. Sustainable implementation requires conceptual clarification, robust evaluations and cross-sectoral structural and funding models. The prevention of the need for long-term care should be given greater focus in health policy and be more firmly embedded within the statutory health and long-term care insurance systems.

## Einleitung

Die Zahl von Menschen im höheren Lebensalter in der Gesellschaft nimmt zu; zugleich korreliert das Alter des Menschen mit dem Risiko, pflegebedürftig zu werden [1]. Eine alternde Bevölkerung, die Zunahme chronischer Erkrankungen sowie begrenzte finanzielle Ressourcen und der Fachkräftemangel stellen das Gesundheits- und Pflegesystem vor erhebliche Herausforderungen. Der Wissenschaftsrat betont in diesem Zusammenhang die Notwendigkeit eines Paradigmenwechsels hin zu einer stärkeren gesellschaftlichen Gesundheitsorientierung sowie zur Förderung von Lebensqualität, Teilhabe und Gesundheitskompetenz [2]. Auch in der Politik wird dieser Handlungsbedarf erkannt. Im Kontext der Anpassung der Pflegeversicherung an die gesellschaftlichen Herausforderungen wird klar betont, dass Prävention, Gesundheitsförderung und Rehabilitation künftig systematischer genutzt werden müssen, um Pflegebedürftigkeit zu vermeiden, zu verzögern und die Selbstständigkeit älterer Menschen möglichst lange zu erhalten [3]. Vor diesem Hintergrund gewinnen insbesondere niedrigschwellige, aufsuchende und präventiv ausgerichtete Ansätze an Bedeutung, wie sie die Präventiven Hausbesuche (PHB) darstellen.

Für PHB liegt in der Literatur bislang keine einheitliche Definition vor [4]. Sie werden unter anderem als „systematische Erfassung individueller Bedarfe älterer, nicht institutionalisierter Menschen im Rahmen eines anlassunabhängigen Hausbesuchs“ beschrieben [5]. Darüber hinaus werden sie über ein breites Zielspektrum definiert, das von der Prävention und Abschwächung von Pflegebedürftigkeit über die Vermeidung von Stürzen bis hin zur Förderung von Lebensqualität, sozialer Teilhabe, Eigenständigkeit, Mobilität und Gesundheitsverhalten reicht, um den Verbleib in der Häuslichkeit zu unterstützen [6, S. 11]. Zudem werden PHB als „Methode zur Früherkennung von Risikofaktoren und Erkrankungen, zur frühzeitigen Intervention bei bestehenden Risikofaktoren und Erkrankungen sowie zur gezielten Beeinflussung von Merkmalen des Lebensstils, der Lebenslage und der Umwelt“ verstanden [7, S. 147]. Laut Nolting und Marschall sind PHB gut dazu geeignet, im Sinne der „Primärprävention von Pflegebedürftigkeit“ [8, S. 38] genutzt zu werden, sofern die Ansprache der Gesamtbevölkerung bei Überschreiten einer festgelegten Altersschwelle proaktiv erfolgt.

Die Definitionen zeigen, dass mit PHB sowohl präventive als auch ressourcenorientierte Ansätze verbunden werden, ebenso wie verhaltenspräventive und lebensweltbezogene Perspektiven. Allen Ansätzen ist gemein, dass die Intervention im Zuhause der älteren Menschen erfolgt und das übergeordnete Ziel verfolgt wird, den Erhalt selbstständiger Lebensführung sowie den Verbleib in der Häuslichkeit zu fördern. Im Sinne des Setting-Ansatzes der Gesundheitsförderung wird das häusliche Umfeld dabei nicht nur als Ort individueller Verhaltensänderung verstanden, sondern als zentraler Lebensweltkontext, in dem gesundheitsrelevante Bedingungen gestaltet und beeinflusst werden können.

Vor dem dargestellten Hintergrund ergeben sich 2 zentrale Fragestellungen:

- warum PHB als Ansatz zur Unterstützung einer selbstständigen Lebensführung im eigenen Zuhause bislang nicht stärker gesundheitspolitisch fokussiert werden,
- wie zeitnah eine nachhaltige Struktur- und Finanzierungsreform in der gesetzlichen Kranken- und Pflegeversicherung (vgl. Maßnahme 1.7.2 Förderung PHB) umgesetzt werden kann, um dauerhaft präventive Ansätze zu ermöglichen.

Um diese Kernfragen diskutieren zu können, wird im Folgenden die Ausgangslage beschrieben, PHB-Angebote in Deutschland werden dargestellt und die Ergebnisse diskutiert. Darüber hinaus werden wissenschaftliche Empfehlungen zur Umsetzung aufgeführt und ein Fazit gezogen.

## Ausgangslage

Der Deutsche Bundestag hat am 18.06.2015 ein Gesetz zur Stärkung der Gesundheitsförderung und der Prävention verabschiedet. Im SGB V wurden unter anderem in Artikel 1 nach § 1 Satz 1 der Zusatz der „… Förderung der gesundheitlichen Eigenkompetenz und Eigenverantwortung der Versicherten“ [9, S. 1368] und der § 20a, „Leistungen zur Gesundheitsförderung und Prävention in Lebenswelten“ [9, S. 1369] ergänzt. PHB sind grundsätzlich an diese Zielrichtungen anschlussfähig, insbesondere die Stärkung von Gesundheitskompetenz, die frühzeitige Identifikation von Risiken sowie die Förderung eines möglichst langen selbstständigen Lebens im Alter. Entsprechend wird ihnen auch auf politischer Ebene ein hohes Potenzial zugeschrieben, etwa im Kontext der Stellungnahme des Bundesrates zum „Gesetz zur Verbesserung der Gesundheitsversorgung und Pflege“ [10], in der eine verzahnte Finanzierung über Altenhilfe und gesetzliche Krankenversicherung (GKV) angeregt wurde.

Dieser Vorschlag der verzahnten Finanzierung durch Leistungen der GKV und der Altenhilfe verweist zugleich auf ein zentrales Strukturmerkmal von PHB in Deutschland. Ursprünglich als medizinisch-risikoorientierte Programme konzipiert, entwickelten sie sich zunehmend zu heterogenen, sozialraumorientierten Ansätzen [11]. Daraus resultiert eine Schnittstellenposition zwischen verschiedenen Leistungsbereichen, insbesondere dem Gesundheitswesen, der Pflege und der kommunalen Altenhilfe. Damit verbunden sind Fragen der Finanzierung, der Zuständigkeit, des Interventionskonzepts und der wissenschaftlichen Evaluation [12], die auf eine fragmentierte Governance-Struktur der PHB in Deutschland hinweisen.

PHB werden seit rund 4 Jahrzehnten international wissenschaftlich untersucht [13]. Frühe Studien aus Dänemark [14, 15] sowie aus der Schweiz [16] zeigen vielversprechende Effekte im Hinblick auf Sterblichkeits- und Institutionalisierungsraten. In Dänemark wurden die Erkenntnisse der ersten Studien früh in politische Praxis überführt: Seit 1998 sind alle Kommunen in Dänemark gesetzlich dazu verpflichtet, PHB 2‑mal im Jahr für Senior*innen, die 75 Jahre und älter sind, anzubieten [15, 17]. Im Laufe der Zeit wurde hier eine Anpassung vorgenommen. Die Mindestaltersgrenze wurde auf 80 Jahre angehoben. In Anspruch nehmen können PHB jedoch alle zwischen 65 und 79 Jahren, die gefährdet oder sozial exponiert sind [18]. Die Hauptziele des dänischen Gesetzes sind die Stärkung des Vertrauens älterer Menschen in das Sozialsystem und das frühzeitige Erkennen eines Hilfebedarfs bei der Zielgruppe [17]. Das Angebot hat damit in Dänemark eine klare gesetzliche Rahmung und institutionelle Verankerung.

Auch für Deutschland deuten verschiedene Projekte in unterschiedlichen Kontexten auf einen anhaltenden gesellschaftlichen Bedarf an PHB und Interesse an entsprechenden Angeboten hin [4, 19, 20]. Kommunale Erfahrungen in Deutschland zeigen, dass präventive Angebote sowie Informations‑, Beratungs‑, Hilfe- und Versorgungsleistungen insbesondere von Menschen mit geringem Einkommen, niedriger formaler Bildung, sozialer Isolation oder mit Migrationshintergrund unzureichend in Anspruch genommen werden. Diese Erfahrungen werden durch Ergebnisse der Versorgungs- und Präventionsforschung gestützt [u. a. 21–23], die auf bestehende Zugangsbarrieren sowie eine sozial ungleiche Inanspruchnahme von Präventions- und Unterstützungsleistungen hinweisen. Hieraus ergibt sich für Kommunen ein erheblicher Handlungsdruck, da bestehende Angebotsstrukturen ausgerechnet diejenigen Zielgruppen nicht erreichen, die einen erhöhten Unterstützungsbedarf aufweisen. Vor diesem Hintergrund gewinnen aufsuchende und sozialraumorientierte Ansätze zunehmend an Bedeutung, da sie das Potenzial haben, auch schwer erreichbare Zielgruppen anzusprechen. Die Wirksamkeit sozialraumorientierter PHB in Deutschland konnte bislang nicht durch kontrolliert-randomisierte Studien bewertet werden. Schulz-Nieswandt et al. [24] führen dies maßgeblich auf Budgetrestriktionen zurück, die die Umsetzung entsprechender Studiendesigns erheblich einschränken.

Die internationale Evidenzlage zu PHB ist insgesamt uneinheitlich: Während einzelne Studien positive Effekte auf spezifische Outcomes zeigen, berichten viele Übersichtsarbeiten keine konsistenten Wirkungen, etwa im Hinblick auf Pflegebedürftigkeit, Lebensqualität oder funktionale Fähigkeiten [15]. Als zentrale Erklärung für diese inkonsistente Befundlage wird in der Literatur die hohe Heterogenität der Interventionen hervorgehoben, sowohl hinsichtlich Zielgruppen, Settings, Intensität, Qualifikation der Durchführenden als auch der zugrunde liegenden Zielgrößen [u. a. 13, 15, 25]. Für Deutschland zeigt Dapp [13] zudem, dass zwar einzelne kontrolliert-randomisierte Studien existieren und in internationale Reviews einfließen, die Evidenzbasis jedoch bislang durch modellhafte, regional begrenzte Projekte und deren Evaluationsdesigns geprägt ist, die keine Wirksamkeitsnachweise im Sinne kontrolliert-randomisierter Studien zulassen.

Vor diesem Hintergrund ist in den vergangenen Jahren eine Vielzahl von PHB-Projekten und -Erprobungen im kommunalen Raum entstanden. Auch das bundesweite Netzwerk „Präventive Hausbesuche“ verdeutlicht diese Entwicklung: Es dokumentiert eine wachsende Verbreitung und trägt zur Vernetzung, zum Wissenstransfer sowie zur Weiterentwicklung von PHB-Konzepten in Deutschland bei [26]. Bislang fehlen eine systematische Erfassung und vergleichende Analyse der unterschiedlichen Ausgestaltungsformen von PHB in Deutschland. Angesichts der beschriebenen konzeptionellen Vielfalt, der fragmentierten Finanzierungs- und Governance-Strukturen sowie der uneinheitlichen Evidenzlage stellt sich daher die Frage, wie PHB in Deutschland konkret ausgestaltet sind, welche Ziele sie verfolgen und inwiefern sich unterschiedliche Konzepttypen identifizieren lassen.

Zur Gewinnung eines Überblicks über bestehende Ansätze für PHB in Deutschland wurde eine explorative Recherche durchgeführt. Der Fokus lag dabei insbesondere auf größeren, von Bundesländern bzw. Stadtstaaten initiierten Programmen bzw. Verstetigungen von PHB der letzten 10 Jahre mit wissenschaftlicher Evaluation. Darüber hinaus wurden einige wenige Einzelprojekte in die Analyse integriert, sofern hierzu ausreichend Informationen, insbesondere evaluative Ergebnisse, vorlagen. Aufgrund der Expertise der Autorinnen im Handlungsfeld wurden zunächst die Internetseiten der bekannten Projekte durchsucht, die Webseite des Bundesinstituts für Öffentliche Gesundheit, auf der auch das bundesweite Netzwerk „Präventive Hausbesuche“ verortet ist, sowie eigene Publikationen hinzugezogen. Ergänzend wurde über Google nach Projekten bzw. deren Evaluations- und Abschlussberichten gesucht. Ein Anspruch auf Vollständigkeit besteht nicht. Die Analyse erfolgte hinsichtlich der Konzepte PHB: die Qualifikation der Hausbesucher*innen, das Ziel, die Zielgruppe, der Zugangsweg, das Leistungsspektrum, die Evaluation, die Hauptergebnisse, Governance/Träger und die Finanzierung des Angebots.

## PHB in Deutschland

In den vergangenen Jahren gab es 9 groß angelegte Projekte mit wissenschaftlicher Evaluation bzw. deren Verstetigungen, die in Tab. 1 dargestellt sind. Darüber hinaus gibt bzw. gab es zahlreiche weitere Angebote. Insgesamt 26 zusätzliche Angebote PHB in Deutschland konnten recherchiert werden: *Amt Bornhöved, Arnsberg, Chemnitz, Cuxhaven,* *Donnersberg, Emlichheim, Flensburg,* *Frankfurt am Main, Hameln, Kamp-Lintfort, Kiel, Kreis Bergstraße (Odenwald), Kreis Heinsberg, Kreis Lippe, Kreis Pinneberg,* *Kreis Rendsburg-Eckernförde, Kreis Steinburg, Landkreis Hameln-Pyrmont, Leipzig,* *Lübeck,* *Magdeburg, München, Neumünster, Osnabrück, Sindelfingen und Stuttgart.*

**Tab. 1:** Groß angelegte Projekte bzw. Verstetigungen von Präventiven Hausbesuchen (PHB) der letzten 10 Jahre mit wissenschaftlicher Evaluation

Name des Angebots und Region, Zeitraum	Qualifikation der Hausbesucher*innen	Ziel, Zielgruppe, Zugangsweg, Leistungsspektrum (LSP)	Evaluation	Hauptergebnisse	Governance/Träger Finanzierung
Programm „Gesund Älter Werden“ (GÄW; [27]) *Niedersachen* 2004–2006 kontrolliert-randomisierte Studie in 10 Stadtteilen Hannovers → direkte Verstetigung Stand 04/2015: Hannover Stadt, Hildesheim, Verden, Braunschweig, Wittingen und Delmenhorst	Geschulte Berater*innen mit Grundqualifikationen im Gesundheitswesen	Verbesserung des Gesundheitsstatus und der Lebensqualität sowie die Erhaltung der Eigenständigkeit; Förderung von Gesundheitsressourcen Zunächst AOK-Versicherte > 63 J., später 50–79 J., aktive Ansprache LSP: Präventive Beratung, Gesundheitsförderung, Vernetzung regionaler Angebote	Wissenschaftliche Begleitung (kontrolliert-randomisierte Studie nach der Intention-to-treat-Methode), Evaluation (Wahl eines anspruchsvollen quasiexperimentellen Studiendesigns), Befragungen und Prä-Post-Analysen innerhalb der Interventionsgruppe für Daten	Effekte der Präventiven Hausbesuche (PHB) waren: pflegepräventiv, geringere Mortalitätsrate der Teilnehmer*innen, geringere Kosten für Pflege, geringeres Sturzrisiko der Teilnehmer*innen. Keine Unterschiede in Bezug auf: Krankenhausfälle und -kosten, Arzneimittel, Herzinfarkt, Schlaganfälle Hohe Aufdeckungsrate behandlungsbedürftiger Risiken und Symptome, Aufdeckung sozialer Problemlagen, pflegepräventive Wirkung ist anzunehmen, von einer Verlängerung der Lebenszeit kann ausgegangen werden, Verbesserung der Lebensqualität (besonderer Gruppen in einzelnen Dimensionen) und des Lebensstils (Impfen, Ernährung, Gewicht, Flüssigkeit etc.), hohe Akzeptanz und Zufriedenheit Für die Verstetigung: Eingrenzung des Angebots auf Menschen im Alter von 63–78 Jahren mit höherem Unterstützungs- und Interventionsbedarf	AOK – Die Gesundheitskasse für Niedersachsen
Landes-Modellprojekt „Prävention für Senioren Zuhause (PräSenZ)“ [28] *Baden-Württemberg* 2014–2017 Neuweiler, Rheinfelden, Ulm	Sozialarbeit/Pflegefachpersonen (begleitende Schulung)	Rahmenprogramm: Entwicklung und Erprobung kommunal anschlussfähiger PHB für selbstständig zu Hause lebende Senior*innen; Konkretisierung in kommunalen Konzepten Ulm: Aufbau kommunaler Beratung unabhängig von Eigeninitiative, sozialem Status und wirtschaftlicher Lage zur Unterstützung selbstständigen Wohnens und der Sozialplanung Senior*innen 75 und 80 Jahre; kommunaler Geburtstagsbrief mit Termin und Telefonnummer für Absage LSP: Information bis Beratung Rheinfelden: Förderung von Lebensqualität, Stärkung der kommunalen Altenhilfe und Unterstützung der Sozialplanung ab dem 75. Lebensjahr; kommunaler Brief mit Ankündigung eines Telefonats LSP: Information bis niedrigschwelliges CM Neuweiler: Kennenlernen der Versorgungsbedarfe sowie Auf- und Ausbau geeigneter Strukturen ab dem 75. Lebensjahr, kommunaler Brief mit Ankündigung eines Telefonats LSP: Information bis niedrigschwelliges CM	Wissenschaftliche Begleitung (Konzeptentwicklung, Implementierung, Evaluation), Evaluation mit Mixed-Methods-Ansatz (Prozess- und Ergebnisevaluation mit qualitativen Interviews/Fokusgruppen und quantitativer Dokumentenanalyse) und Abschlussbericht	PHB konnten in allen Kommunen umgesetzt werden; Erreichbarkeit/Akzeptanzquote^a^ in Abhängigkeit von Zugangswegen: Ulm: Akzeptanzquote: 51 % Rheinfelden: Akzeptanzquote: 13 % Neuweiler: Akzeptanzquote: 47 % LSP abhängig von den kommunalen Angebotsstrukturen. Besonderheit Neuweiler (kleine Kommune) eher Bedarf für Entlastung der Angehörigen, daher Einstellung der PHB und Aufbau einer Tagespflege Senior*innen: Informationsgewinn, aktive Ansprache von Themen und Sensibilisierung, Ansprechpartner in den Kommunen, höheres Sicherheitsgefühl Kommunen: Bekanntheit des kommunal vorgehaltenen Angebotes verbessert, Imagegewinn	Rahmenprogramm: kommunale Steuerung Finanzierung Modellprojekt: Land Baden-Württemberg und gesetzliche und private Pflegekassen über §§ 45c und 45d SGB XI
Projekt „PräSenZ im Quartier (PIQ)“ [29] *Ulm *[30] und *Rheinfelden* [31] 2014–2019 → Verstetigung	Sozialarbeit/Pflegefachpersonen	Erreichung vulnerabler älterer Menschen, Förderung der Sozialraumentwicklung sowie des Aufbaus nachhaltiger organisatorischer und finanzieller Strukturen zur Verstetigung PHB Ulm: Senior*innen 75 Jahre; kommunaler Geburtstagsbrief mit Termin und Telefonnummer für Absage LSP: wie in PräSenZ Rheinfelden: ältere Menschen mit Unterstützungsbedarf; quartiersbezogene Ansprache; Zugangsweg: Multikanalstrategie LSP: Information bis niedrigschwelliges CM, inkl. Betreuung	Wissenschaftliche Begleitung (Implementierung, Evaluation), Evaluation mit Mixed-Methods-Ansatz (Prozess- und Ergebnisevaluation mit qualitativen Interviews und quantitativer Dokumentenanalyse) und Abschlussbericht	Erreichbarkeit und Zielgruppen können über die Zugangswege gesteuert werden: proaktive Ansätze erreichen ein breites Spektrum, inkl. Menschen mit Migrationshintergrund, Vermittlung über Kooperationspartner besonders vulnerable Gruppen Senior*innen: Frühzeitige Sensibilisierung, Informationsgewinn, Erhöhung Zugangschancen zu Unterstützungsleistungen und Hilfen; Stabilisierung vulnerabler Lebenslagen niedrigschwelliges Case Management erforderlich Sozialraumentwicklung: Notwendig sind definierte Schnittstellen für kommunale Kooperation und Vernetzung, höhere politische Aufmerksamkeit für Seniorenarbeit	Kommunale Steuerung aufbauend auf PräSenZ Finanzierung: Projektphase: § 45c SGB XI und kommunale Mittel Verstetigung: Haushaltsmittel
Gemeindeschwester^plus^ [32] *Rheinland-Pfalz* 2015–2018 Modellphase in 9 Kommunen [24] 2019–2022 Verstetigungsphase in 26 Kommunen [33] → seit 2023 Verstetigung als flächendeckendes Landesprogramm	Modellphase: Pflegefachpersonen (begleitende Schulung) Verstetigungsphase: Pflegefachpersonen Verstetigung: 90 Pflegefachpersonen fest angestellt (keine Aussage zu Vollzeitäquivalenten)	Modellphase: Menschen ab 80 J., ohne Pflegegrad; Multikanalstrategie (kommunale Entscheidung) LSP: Information bis niedrigschwelliges CM Verstetigungsphase: Fokus Einsamkeit LSP: Information bis niedrigschwelliges CM Verstetigung: Förderung eines selbstständigen und selbstbestimmten Lebens zu Hause Ältere Menschen (keine Altersgrenze) LSP: Beratung, Vermittlung, präventive Unterstützung und niedrigschwellige Begleitung	Modellphase 2015–2018: externe Mixed-Methods-Prozess- und Ergebnisevaluation Verstetigungsphase: 2019–2022 Externe Mixed-Methods-Prozess- und Ergebnisevaluation (modular aufgebaute Prozess- und Ergebnisevaluation) und Ergebnisbericht	Modellphase: Bedeutung von Vernetzung, Rollenklärung und kommunaler Einbettung für gelingende Implementierung; Bekanntheit des Angebots bei Bürger*innen: 55 % (Marktplatzinterviews) Zentrale Themen: Aktivierung, Gesellung und Hilfebedarf, ca. ein Drittel der Anfragenden oder Beratenen wurde an den Pflegestützpunt vermittelt Initiierung von ca. 70 neuen Angeboten in den Kommunen (unter anderem Bewegung, Teilhabe, Mobilität, Erhöhung von Sicherheit, Fachvorträge) Erreichbarkeit: keine Aussage Implementierung gelungen, Akzeptanz in den kommunalen Strukturen vorhanden Verstetigungsphase: Senior*innen: fühlen sich sicherer (59 %), Leben zu Hause hat sich vereinfacht (87 %), Nutzen weiterführende Angebote (64,8 %, davon 25 % Pflegestützpunkt), besser informiert (78 %), Unterstützung bei medizinischen Problemen (43 %); mehr Kontakt mit Gleichgesinnten, Verbesserung der Kommunikation mit den Angehörigen	Rahmenprogramm: landesgesteuert Modellprojekt: Kommunen und Wohlfahrtsverbände Verstetigungsphase: Überwiegend Kommunen, ergänzt durch Wohlfahrtsverbände Finanzierung Modellphase: Landesmittel; Finanzierung Verstetigungsphase: Landesmittel, GKV und Krankenkassenverbände (350.000 € GKV und KKV) Verstetigung: Doppelhaushalt 3,8 Mio. € (2025/2026)
Landesprogramm „Lebendige Quartiere“: Programmschwerpunkt Präventive Hausbesuche [34, 35] *Bremen* (Stadtteil Vahr) und *Bremerhaven* (Quartiere Grünhöfe, Goethestraße, Klushof, Twischkamp und Leherheide West) Bremen: 07/2023–12/2023 → Verstetigung Stadt Bremen seit 01.01.2026 Bremerhaven: 03/2023–12/2023	Bremen: 7 Teilzeitstellen (Sozialarbeit und unbekannt) Bremerhaven: 1 Pflegekraft und Sozialpädagogin hauptamtlich (1 Stelle Vollzeitäquivalent) Kein einheitliches Schulungskonzept	Teilhabe verbessern, Einsamkeit vermeiden, Pflegebedürftigkeit hinauszögern 70-/80-Jährige in eigener Häuslichkeit; quartiersbezogene Ansprache Bremerhaven: Senior*innen 70 Jahre; kommunaler Geburtstagsbrief mit Termin, der abgesagt, verschoben oder ignoriert werden kann, Auswahl der Ortsteile basiert auf ihrem sozioökonomischen Status. Zusätzlich können sich alle Menschen ab 55 Jahren stadtweit bei dem Angebot melden und einen PHB bzw. eine Beratung bekommen LSP beide Kommunen: aufsuchende Beratung und Vermittlung	Bislang keine publizierte wissenschaftliche Evaluation	Zwischenergebnis: hohe Akzeptanz, ältere Menschen fühlen sich „gesehen“	Bremen: Steuerung: Sozialreferat und Umsetzung: freie Träger Bremerhaven: Steuerung und Koordination durch Stabsstelle für Senior*innen im Sozialreferat in Abstimmung mit dem Sozialamt Finanzierung: Landesmittel Verstetigung: in Bremerhaven, solange die Landesmittel verfügbar sind
Berliner Hausbesuche (BHB; [36]) *Berlin* 05/2021–07/2022 Modellphase 1 (2 Bezirke) 11/2022–09/2023 Modellphase 2 (7 Bezirke) → seit 2024 flächendeckende Ausweitung und Verstetigung	Lots*innen Sozial/Pflege/Gesundheit (zusätzliche Schulung) 03/2025: 24 Lots*innen (18 Vollzeitäquivalent)	Lotsenfunktion, Information und Orientierung, Abbau von Zugangsbarrieren Menschen ab 70 J., freiwillige Informationsgespräche zu Hause oder im Sozialraum Multikanalstrategie (Geburtstagsanschreiben ohne Termin, Öffentlichkeitsarbeit und Multiplikator*innen) LSP: Information bis Beratung	Wissenschaftliche Begleitung (Konzeptentwicklung, Implementierung und Evaluation), Evaluation mit Mixed-Methods-Ansatz und Abschlussbericht	Evaluation der 2. Modellphase: Erreichbarkeit durch Öffentlichkeitsarbeit am höchsten, Rückmeldung zur Altersgruppe 70 Jahre gemischt (Altersdurchschnitt 78,1 Jahre) Senior*innen: hohe Zufriedenheit mit Gespräch, Erwartungen erfüllt 78 %, Nützlichkeit des PHB 73 % (sehr nützlich, eher nützlich, Befragung nach mehreren Monaten), Weiterempfehlung 95 % (kurzfristige Befragung) Rückmeldung zu Angebotslücken, z. B. Mobilitätshilfen, Ärzt*innen, die Hausbesuche durchführen, mangelnde „Willkommenskultur“ in bestehenden Angeboten Reibungslose Integration in die Bezirke durch bezirkliche Kooperationspartner	Konzept: landesgesteuert Umsetzung: freie Träger (Malteser) Koordination: Bezirke Finanzierung: Land Berlin 2023: 800.000 € 2025: 1,3 Mio. € (beantragt)
Modellprojekt „Präventive Hausbesuche“ [37] *Niedersachsen* 01/2021–12/2023 Braunschweig, Hameln, Zetel → seit 02/2024 Verstetigung in Braunschweig und 12/2024 in Hameln	Modellprojekt: Braunschweig: 35 geschulte Ehrenamtler*innen Hameln: 2 akademisch ausgebildete Fachkräfte mit Masterabschluss im Bereich der sozialen Arbeit sowie Sozial- und Organisationspädagogik Zetel: 2 Pflegefachpersonen	Unterstützende und koordinierende Angebote zum längeren Verbleib zu Hause Menschen ab 80 J., nicht pflegebedürftig; kostenlos, freiwillig Multikanalstrategie: persönliches Anschreiben, Öffentlichkeitsarbeit LSP: Information bis niedrigschwelliges CM	Wissenschaftliche Begleitung (Konzeptentwicklung, Implementierung, Evaluation), Evaluation mit Mixed-Methods-Ansatz (Prozess- und Ergebnisevaluation mit qualitativen Interviews und quantitativer Dokumentenanalyse) und Abschlussbericht	Akzeptanz sehr hoch und durchweg positiv Wirksamkeit kennzeichnete sich durch 3 Phasen: I. Phase: Gefühl von Unsicherheit prägnant, dies zeigte die hohe Relevanz des niedrigschwelligen Beratungs- und Begleitkonzepts für die Senior*innen auf II. Phase: Gefühl von Wohlbefinden, da Wissen verfügbar gemacht wurde, Entscheidungen durch die Senior*innen selbst getroffen werden konnten; die Gesundheit wurde durch die Beratungen und Empfehlungen positiv beeinflusst, das Gefühl der Zufriedenheit gesteigert III. Phase: große Dankbarkeit für das Angebot und somit für das Kümmern um die Senior*innen; Gefühl der Sicherheit, jederzeit an Informationen zu gelangen und eine*n Ansprechpartner*in vor Ort zu haben Aufgrund dieses erlebten Prozesses ist der Wunsch der Senior*innen nach einer Verstetigung zentral	Rahmenprogramm: landesgesteuert Konzept: kommunale Steuerung Modellprojekt: Land Niedersachsen und kommunale Mittel Verstetigung: Stadt Braunschweig und Stadt Hameln aus Haushaltsmitteln
Hamburger Hausbesuch für Seniorinnen und Senioren [38, 39, 42] *Hamburg* 2018–2019 Pilotphase 1 (Harburg und Eimsbüttel) 2020–2021 Pilotphase 2 (alle Bezirke) 2022–2025 Pilotphase 3 → seit 2025 Verstetigung	Honorarkräfte aus dem Sozial‑/Gesundheitswesen (zusätzliche Schulung)	Förderung aktiver und selbstständiger Lebensführung in der eigenen Häuslichkeit, Präventions- und Unterstützungsbedarfe frühzeitig zu erkennen sowie Vereinsamung im Alter zu vermeiden Menschen ab 80 J.; Geburtstagsanschreiben mit Terminangebot LSP: Information, Beratung, Lotsenfunktion	Zeitraum 2018 bis 2022: Evaluation des Zeitraums mit Mixed-Methods-Ansatz Pilotphase 3: interne standardisierte Nachbefragung (nach 8 Wochen)	Pilotphase 1 und 2: hohe Erreichbarkeit: ca. 35 % Akzeptanzquote Befragung Senior*innen: sehr hohe Zufriedenheit mit dem Gespräch Erwartungen an den Besuch erfüllt: 90 %, Nützlichkeit des Angebots für eigene Situation: 72 % (nützlich, sehr nützlich), geringe Vermittlungsquote und Inanspruchnahme von empfohlenen Angeboten Pilotphase 3: sehr hohe Zustimmung zu Vorgehen und Angebot. Positive Bewertung Schreiben und Termin: 98 %, Weiterempfehlen: 89 %, Empfehlung passgenau: 83 %, empfohlenes Angebot in Anspruch genommen: 21 %, Absicht, empfohlenes Angebot in Anspruch zu nehmen: 62 %, Aufbewahrung Materialien: 96 %	Kommunale Steuerung; externer Auftragnehmer als beliehene Stelle (Übertragung kommunaler Aufgaben) Finanzierung: Freie und Hansestadt Hamburg
Programm „AGATHE – Älter werden in der Gemeinschaft“ [40] *Freistaat Thüringen* 2021 Pilotphase (18 Landkreise und kreisfreie Städte) → Gesetzliche Verankerung zur flächendeckenden Etablierung vorgesehen	54 Fachkräfte mit sozial- oder gesundheitsbezogener Ausbildung (z. B. Sozialarbeit/-pädagogik oder Pflegefachpersonen; keine Aussage zu Vollzeitäquivalenten)	Vermeidung von Einsamkeit, Förderung sozialer Teilhabe und selbstständigen Wohnens Menschen über 63 J., alleinlebend; Multikanalstrategie LSP: Beratung, Begleitung, Vermittlung	Externe Evaluation, Evaluator zugleich teilweise Arbeitgeber von AGATHE-Fachkräften Qualitative Querschnittsevaluation ohne Kontrollgruppe	Senior*innen: vertrauenswürdige Anlaufstelle, Hinweise zu subjektiv wahrgenommener Verringerung von Einsamkeit und Teilhabeaktivierung sowie zu gesteigertem psychischen Wohlbefinden und besserer Lebensqualität; Unterstützung Antragswesen Erreichte Zielgruppe: eher vulnerabel Feststellung von Angebotslücken, Weitergabe an Sozialplanung Bessere Vernetzung von Akteuren und Angeboten, Initiierung neuer Angebote und Veranstaltungen; Bindeglied zu den Netzwerkpartnern sowie zu Verwaltungsstrukturen (insbesondere bei Ansiedlung beim Kreis) Direktere Zugangswege haben höhere Resonanz gezeigt; Öffentlichkeitsarbeit für Vertrauensaufbau	Rahmenprogramm: landesinitiiertes Programm Umsetzung: Kommunen und freie Träger Finanzierung: Pilotphase: Landesförderprogramm Thüringen (ca. 2,2 Mio. €) mit kommunalem Eigenanteil (10–20 %), ggf. ergänzt durch Bundes‑/EU-Mittel Verstetigung: Doppelhaushalt 4,8 Mio. € und 5,9 Mio. (2026 und 2027)

^a^ Akzeptanzquote: Anteil der durchgeführten Hausbesuche an der Anzahl der versendeten Anschreiben.

Tab. 1 verdeutlicht die Heterogenität der PHB-Konzepte und ihrer Governance-Strukturen. Die Analyse der Konzepte erfolgte hinsichtlich folgender Merkmale: Qualifikation der Hausbesucher*innen, Ziel, Zielgruppe, Zugangsweg, Leistungsspektrum, Evaluation, Hauptergebnisse, Governance/Träger und Finanzierung des Angebots. Neben kommunal gesteuerten Angeboten finden sich insbesondere landesinitiierte Rahmenprogramme mit lokaler Umsetzung durch Kommunen, Wohlfahrtsverbände oder freie Träger. Insgesamt zeigt sich eine starke kommunale Einbindung mit unterschiedlich ausgeprägter Konzept- und Steuerungszentralisierung. Ein wiederkehrendes Ergebnis über die Projekte hinweg ist die zunächst überwiegend projektförmige und zeitlich befristete Finanzierung der Angebote. Dies führt dazu, dass Angebote teilweise wieder eingestellt werden oder eine langfristige Verstetigung erschwert ist.

Tab. 1 zeigt darüber hinaus, dass die Zielsetzungen der PHB-Angebote sowohl auf individueller als auch auf struktureller Ebene ansetzen. Auf individueller Ebene stehen insbesondere der Erhalt von Selbstständigkeit, die Förderung sozialer Teilhabe, Prävention von Einsamkeit sowie Information, Beratung und Vermittlung im Vordergrund. Zudem sollen die Zugangschancen zu bestehenden Unterstützungs‑, Versorgungs- und Freizeitangeboten erhöht werden. Auf struktureller Ebene zielen mehrere Projekte zusätzlich auf den Aufbau kommunaler Vernetzungsstrukturen, die Unterstützung sozialräumlicher Entwicklung sowie die Identifikation lokaler Versorgungsbedarfe ab.

Die dargestellten Angebote unterscheiden sich deutlich im Umfang ihres Leistungsspektrums. Dieses reicht von niedrigschwelliger Informationsweitergabe über die Vermittlung in weiterführende Freizeit- und Unterstützungsangebote und fachlich fundierte Beratung bis hin zu Formen eines niedrigschwelligen Case Management für Personen mit komplexen Bedarfslagen. Damit bilden die untersuchten Konzepte ein Kontinuum unterschiedlicher Interventionsintensitäten ab, das jeweils an die lokalen Strukturen, Zielgruppen und Ressourcen angepasst ist. In inhaltlicher Hinsicht adressieren die Hausbesuche vor allem Themen wie gesundheitliche Situation, Prävention (z. B. Bewegung und Ernährung), soziale Teilhabe sowie Information und Beratung zu Unterstützungsangeboten [4, 28, 37, 41]. Auf diese Weise werden sowohl Risikofaktoren wie soziale Isolation, Mangelernährung oder Sturzgefahr frühzeitig erkannt als auch entsprechende Unterstützungsprozesse angestoßen. Eng verbunden mit dem Leistungsspektrum sind die Zielgruppen und Zugangswege der Angebote. Neben einer breiten Ansprache älterer Menschen rücken insbesondere Personen mit eingeschränkten Ressourcen, komplexen Bedarfslagen oder erschwertem Zugang zu bestehenden Unterstützungsstrukturen in den Fokus. Die beschriebenen Zugangswege reichen von proaktiven Geburtstagsanschreiben mit Terminangebot, etwa in Ulm [30] und Hamburg [38], bis hin zu Multikanalstrategien unter Einbezug von Öffentlichkeitsarbeit und Kooperationspartnern [24, 31, 37]. Die Evaluationen zeigen Hinweise, dass proaktive, breit angelegte Ansätze ein breites Spektrum an älteren Menschen erreichen und dazu beitragen können, auch Zielgruppen zu erreichen, die als schwer erreichbar gelten, z. B. Menschen mit Migrationshintergrund. Reaktive Zugangswege, beispielsweise die Zusammenarbeit mit Kooperationspartnern oder die gezielte Ansprache, erreichen eher vulnerable Gruppen mit komplexen Bedarfslagen [12, 33]. Zugänge, die hingegen auf Eigeninitiative der Adressierten beruhen, erreichen eher Personen mit bereits vorhandener Informations- und Handlungskompetenz [29]. Insgesamt wird deutlich, dass Zugangswege nicht nur die Inanspruchnahme beeinflussen, sondern auch die Art der erreichten Bedarfe strukturieren und somit eine gezielte Steuerung zwischen breiter Prävention und gezielter Unterstützung ermöglichen.

Ein weiteres zentrales Ergebnis ist die hohe Bedeutung kommunaler Einbindung und von Netzwerkstrukturen. PHB sind in der Regel eng in bestehende lokale Unterstützungs- und Versorgungsstrukturen eingebettet. Kooperationen mit Akteuren des Gesundheits- und Sozialwesens sowie eine sozialräumliche Vernetzung stellen wesentliche Voraussetzungen für die Umsetzung dar und ermöglichen eine bedarfsgerechte Weitervermittlung und Unterstützung [4, 19, 37]. Hinsichtlich der Umsetzung zeigt sich zudem, dass die Qualifikation der Besuchskräfte stark variiert und von ehrenamtlichen oder geschulten Honorarkräften bis hin zu Fachkräften aus dem Sozial- und Gesundheitswesen reicht. Mit zunehmender Interventionsintensität steigt in der Regel auch der Bedarf an fachlicher Qualifikation.

Die vorliegenden Evaluationen liefern darüber hinaus Hinweise auf mögliche Wirkungen von PHB auf verschiedene Outcome-Dimensionen. In mehreren Projekten zeigen sich positive Entwicklungen im Hinblick auf die soziale Teilhabe älterer Menschen sowie deren Einbindung in lokale Unterstützungsstrukturen. Zudem berichten Evaluationen von Verbesserungen im psychosozialen Wohlbefinden, etwa in Form eines reduzierten Einsamkeitsempfindens sowie eines gesteigerten Gefühls, gesehen und unterstützt zu werden [4, 23, 40]. Darüber hinaus deuten die Ergebnisse darauf hin, dass PHB zur Stärkung der individuellen Gesundheits- und Versorgungskompetenz beitragen und den Zugang zu bestehenden Hilfs- und Beratungsangeboten erleichtern können [4, 32]. Im Sinne gesundheitsfördernder Sozialraumentwicklung zeigen die Ergebnisse zudem, dass PHB nicht nur auf individueller Ebene wirken, sondern auch Impulse für sozialräumliche und kommunale Entwicklungsprozesse setzen können. Mehrere Projekte und Angebote berichten von Rückkopplungseffekten aus den PHB in kommunale Verwaltungs- und Netzwerkstrukturen, beispielsweise durch die Identifikation von Angebotslücken oder bislang ungedeckten Unterstützungsbedarfen. Einzelne Konzepte, wie etwa die Gemeindeschwester^plus^, umfassen darüber hinaus die Initiierung und Durchführung gesundheitsfördernder Angebote im Sozialraum [33, 37]. Neben diesen strukturellen Effekten zeigen sich auch Hinweise auf Wirkungen im Hinblick auf subjektive Sicherheit und kommunale Sichtbarkeit. So berichten einzelne Evaluationen von einem erhöhten Sicherheitsgefühl älterer Menschen, insbesondere durch das Wissen, bei Bedarf auf verlässliche Ansprechpartner zurückgreifen zu können. Zudem können PHB zu einer höheren Sichtbarkeit kommunaler Unterstützungsangebote sowie zu einem Imagegewinn der Kommunen beitragen.

Bei den Evaluationen der dargestellten Angebote handelt es sich überwiegend um projektbezogene Prozess- und Ergebnisevaluationen mit qualitativen oder Mixed-Methods-Designs. Kontrollierte oder randomisierte Studiendesigns finden sich nicht. Teilweise beruhen die Ergebnisse auf externen Evaluationen, teilweise auf Evaluationen im Rahmen der wissenschaftlichen Begleitung oder der programmeigenen Evaluationen. Die Aussagekraft der berichteten Effekte ist daher insgesamt eingeschränkt. Kausale Rückschlüsse auf die Wirksamkeit von PHB sind nicht möglich. Zudem erschwert die Heterogenität der Konzepte, Zielgruppen, Ergebnisdimensionen und Evaluationsmethoden eine vergleichende Bewertung der Ergebnisse.

## Diskussion und Empfehlungen

PHB haben sich zu einem flexibel an lokale Bedarfe, Zielgruppen und Versorgungsstrukturen anpassbaren Konzept entwickelt. Die Schwerpunktsetzung erfolgt entlang regionaler und kommunaler Bedarfe sowie bestehender Angebotsstrukturen.

PHB können über ihre Funktion als Informations- und Beratungsangebot hinaus eine Rolle in der frühzeitigen Bedarfserkennung und Steuerung im Versorgungssystem einnehmen [4, 29, 36, 37]. Gleichzeitig zeigt sich ein strukturelles Spannungsfeld: Obwohl PHB überwiegend kommunal organisiert sind, verfolgen sie gesundheits- und pflegebezogene Zielsetzungen, die insbesondere im SGB V und SGB XI verankert sind. Daraus ergibt sich ein Ungleichgewicht zwischen kommunaler Verantwortung und den rechtlichen und finanziellen Handlungsmöglichkeiten. Aktuelle gesundheitspolitische Entwicklungen, insbesondere die stärkere Ausrichtung auf Prävention, kommunale Versorgung und erweiterte pflegerische Kompetenzen, könnten die strukturelle Verankerung von PHB künftig begünstigen.

Die Evidenzlage weist darauf hin, dass PHB grundsätzlich wirksam sein können, insbesondere hinsichtlich sozialer Teilhabe, psychosozialen Wohlbefindens und verbesserter Zugänge zu Unterstützungsangeboten. Zugleich bestehen jedoch weiterhin Unsicherheiten hinsichtlich spezifischer Wirkmechanismen und Erfolgsdeterminanten [13]. Dies ist unter anderem auf die hohe Heterogenität der Konzepte, Zielgruppen und Umsetzungsformen und die damit verbundenen methodischen Herausforderungen in der Evaluation zurückzuführen.

**Abb. 1:**
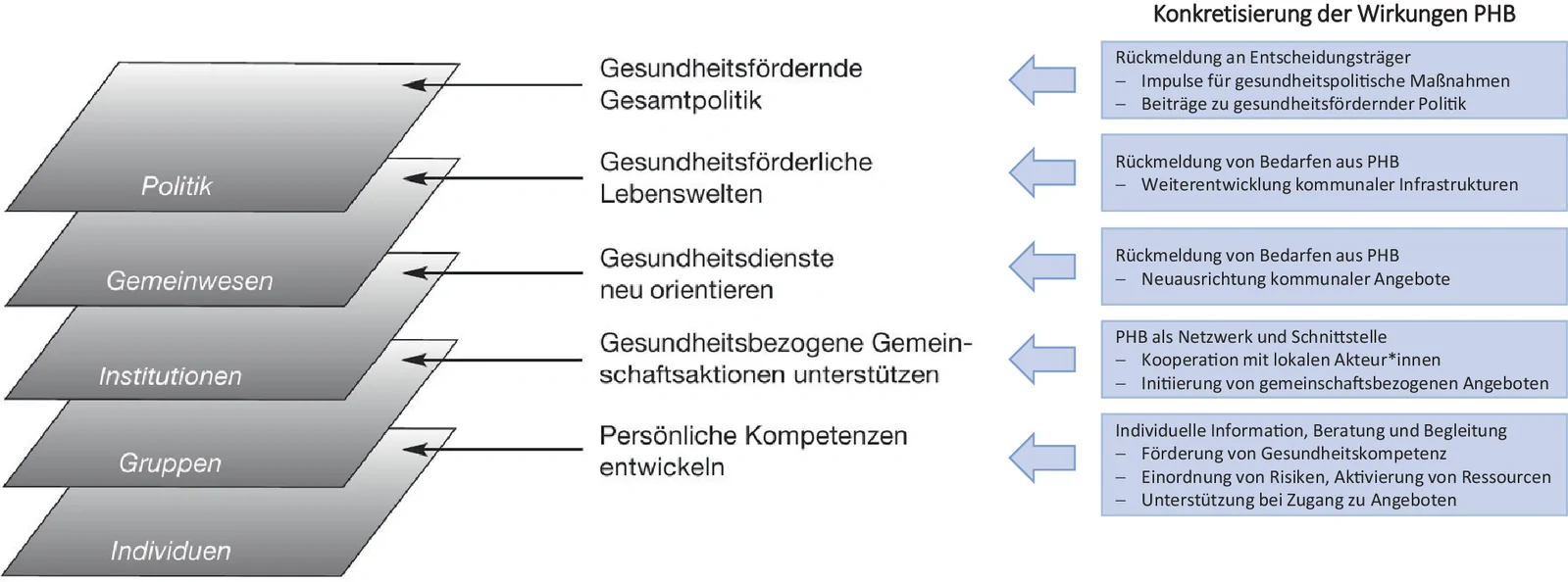
Einordnung Präventiver Hausbesuche (PHB) in das Mehrebenenmodell der Gesundheitsförderung von Kaba-Schönstein ([43], mit freundlicher Genehmigung der Autorin)

Abb. 1 ordnet PHB in das Mehrebenenmodell der Gesundheitsförderung von Kaba-Schönstein [42] ein und verdeutlicht mögliche Einflussbereiche auf individueller, sozialräumlicher und struktureller Ebene. PHB übernehmen dabei gleichzeitig gesundheitsfördernde, präventive, vermittelnde und begleitende Funktionen auf unterschiedlichen Ebenen der Gesundheitsförderung. Sozialräumlich orientierte PHB verbinden Gesundheitsförderung, Prävention und niedrigschwelliges Case Management. Insbesondere bei höherer Interventionsintensität verschwimmen die Grenzen zwischen Gesundheitsförderung, universeller Prävention und bedarfsorientierter Versorgung zunehmend.

PHB bewegen sich dabei in einem Spannungsfeld zwischen populationsbezogener Prävention und zielgruppenspezifischer Unterstützung, wie es im Sinne des Präventionsparadoxons [4] beschrieben wird. Während proaktive Zugangswege breite Bevölkerungsgruppen erreichen, adressieren intensivere oder stärker netzwerkbasierte Ansätze eher vulnerable Personen mit komplexen Bedarfslagen.

PHB erscheinen damit nicht nur als isolierte Einzelintervention, sondern vielmehr als Teil kommunaler Infrastrukturentwicklung im Alter. Ihre Bedeutung liegt nicht ausschließlich in individuellen Beratungs- oder Informationsleistungen, sondern auch in ihrer Funktion als Schnittstelle zwischen älteren Menschen, kommunalen Unterstützungsstrukturen sowie Akteuren des Gesundheits- und Sozialwesens. Dadurch entsteht ein Rückkopplungsmechanismus zwischen individuellen Bedarfslagen und kommunaler Sozialraumgestaltung.

PHB intervenieren an einer Schnittstelle institutioneller und sektoraler Zuständigkeiten und orientieren sich an einem multidimensionalen Modell selbstständiger Lebensführung, wie sie dem Modell der International Classification of Functioning, Disability and Health (ICF) zur funktionalen Gesundheit zugrunde liegt [44]. Diese sektorübergreifende und lebensweltorientierte Perspektive stellt ein wesentliches Potenzial der PHB dar, zugleich aber auch eine strukturelle Herausforderung innerhalb der fragmentierten Finanzierungs- und Zuständigkeitsstrukturen.

Vor diesem Hintergrund können sozialräumlich orientierte PHB als komplexe Interventionen verstanden werden, bei denen unterschiedliche Akteure, Zielgrößen und Interventionselemente ineinandergreifen und Wechselwirkungen zwischen Intervention und sozialem Kontext entstehen [45]. Die Ergebnisse legen daher nahe, PHB stärker als kommunale Interventionssysteme zu verstehen und nicht primär als Intervention zur Reduzierung der Pflegeprävalenz. Klassische Wirksamkeitsnachweise im Sinne kontrolliert-randomisierter Studiendesigns stoßen hier an Grenzen. Gerade für sozialraumorientierte PHB erscheint ein theoriegeleiteter und multiperspektivischer Evaluationsansatz sinnvoll, der sowohl Wirkungen auf individueller Ebene als auch strukturelle Veränderungen im Sozialraum erfasst. Hierfür sind neben der Finanzierung der Interventionen selbst auch Ressourcen für belastbare Evaluationen erforderlich.

Ein weiterer zentraler Aspekt betrifft die Nachhaltigkeit von PHB. Die Ergebnisse zeigen, dass viele Angebote projektförmig und zeitlich befristet umgesetzt werden, obwohl insbesondere sozialraumorientierte PHB auf längerfristige strukturelle Veränderungen abzielen. Daher bedarf es langfristiger Entwicklungs- und Finanzierungsperspektiven. Verstetigungsstrategien sollten deshalb bereits in der Konzeptionsphase mitgedacht werden.

Aus der dargestellten Analyse lassen sich folgende zentrale Handlungsempfehlungen für die Weiterentwicklung und nachhaltige Implementierung PHB ableiten. Entlang des Mehrebenenmodells der Gesundheitsförderung betreffen diese sowohl die individuelle als auch die sozialräumliche und strukturelle Ebene, zusätzlich werden Empfehlungen zum Evaluationsdesign formuliert.

### Individuelle Ebene

- Zugangswege sollten zielgruppenspezifisch ausgestaltet werden, um sowohl breite Bevölkerungsgruppen als auch vulnerable ältere Menschen zu erreichen.
- Die Qualifikation der Besuchskräfte sollte sich an der Interventionsintensität und den Anforderungen der jeweiligen Zielgruppen orientieren.
- Die Ziele sollten an Umfang und Intensität der Intervention angepasst werden.

### Sozialräumliche Ebene

- Die kommunale Vernetzung mit Akteuren des Gesundheits‑, Pflege- und Sozialwesens sollte als zentrale Voraussetzung für die Umsetzung von PHB systematisch gestärkt werden.
- PHB sollten als Bestandteil gesundheitsfördernder Sozialraumentwicklung verstanden und eng an bestehende kommunale Unterstützungsstrukturen angebunden werden.

### Strukturelle Ebene

- Für die Umsetzung von PHB sollten nachhaltige und sektorübergreifende Finanzierungsmodelle entwickelt werden, die kommunale Altenhilfe, Gesundheitswesen und Pflege stärker miteinander verzahnen.
- Sozialräumlich orientierte PHB sollten als komplexe Interventionen mit theoriegeleiteten und multiperspektivischen Evaluationsansätzen wissenschaftlich begleitet werden.
- Verstetigungsstrategien sowie Ressourcen für wissenschaftliche Begleitung und Evaluation sollten bereits in der Konzeptionsphase eingeplant werden.

### Evaluationsdesign

- Evaluationsdesigns sollten der Komplexität sozialräumlicher PHB Rechnung tragen und neben individuellen Outcomes auch strukturelle und sozialräumliche Effekte berücksichtigen.
- Ressourcen für wissenschaftliche Begleitung und langfristige Evaluation sollten bereits in der Konzeptions- und Förderphase der Angebote verbindlich eingeplant werden.

Bereits vorliegende wissenschaftliche Ergebnisse sollten bei der Ausgestaltung der Konzepte von PHB zwingend berücksichtigt werden.

## Fazit

Die Analyse zeigt, dass PHB gegenwärtig ein heterogenes und kontextabhängig ausgestaltetes Instrument der kommunalen Seniorenarbeit an der Schnittstelle von Gesundheitsförderung, Prävention, sozialer Teilhabe und kommunaler Daseinsvorsorge darstellen. Eine einheitliche Definition des Konzepts, die über die aufsuchende Durchführung im häuslichen Umfeld und die präventive Orientierung hinausgeht, liegt bislang nicht vor. Dies erschwert sowohl die Vergleichbarkeit der Ansätze als auch die Transparenz gegenüber den Zielgruppen [4].

Hinsichtlich der Evaluation zeigt sich ein Bedarf an methodisch angemessenen, an die Komplexität und Heterogenität der Intervention angepassten Ansätzen, die sowohl soziale als auch versorgungsbezogene Effekte angemessen abbilden können [13, 46]. Gleichzeitig erscheint im Anschluss an Schulz-Nieswandt et al. [24] die Frage weniger „Ob?“ als vielmehr „Wie?“ zentral für die Umsetzung PHB zu sein. Entscheidend sind die Ausgestaltung der Angebote, ihre kommunale Einbettung sowie verlässliche Finanzierungs- und Verantwortungsstrukturen für Reichweite, Akzeptanz und Nachhaltigkeit. Für eine langfristige Implementierung sind konzeptionelle Klärungen und belastbare Evaluationen erforderlich.

Die derzeit vielfach projektförmige Umsetzung sowie die Verortung an der Schnittstelle verschiedener Sozialgesetzbücher verdeutlichen zudem den Bedarf an einer stärkeren strukturellen und rechtlichen Verankerung der PHB [8]. Die gezielte Schulung der Berater*innen, bspw. als Community Health Nurse (CHN) als Spezialisierung der Advanced Practice Nurse (APN) für den kommunalen Bereich, um die Gesundheit und medizinische Versorgung direkt vor Ort in den Stadtteilen oder ländlichen Gemeinden zu fördern [47], ist erst mit einer verlässlichen Finanzierung und der Integration PHB in feste Versorgungsstrukturen zielführend.

## Data Availability

Alle dieser Arbeit zugrunde liegenden Daten sind in diesem Artikel enthalten.
